# The functional and structural neural correlates of dynamic balance impairment and recovery in persons with acquired brain injury

**DOI:** 10.1038/s41598-022-12123-6

**Published:** 2022-05-14

**Authors:** Katherin Joubran, Simona Bar-Haim, Lior Shmuelof

**Affiliations:** 1 Department of Cognitive and Brain Sciences, Ben-Gurion University of the Negev, P.O. Box 653, 84105 Beer-Sheva, Israel; 2 Zlotowski Center for Neuroscience, Ben-Gurion University of the Negev, 84105 Beer-Sheva, Israel; 3 Department of Physical Therapy, Zefat College, Zefat, Israel; 4 Department of Physical Therapy, Recanati School for Community Health Professions, Ben-Gurion University of the Negev, Beer Sheva, Israel

**Keywords:** Basal ganglia, Cerebellum, Motor cortex, Rehabilitation

## Abstract

Dynamic balance control is associated with the function of multiple brain networks and is impaired following Acquired Brain Injury (ABI). This study aims to characterize the functional and structural correlates of ABI-induced dynamic balance impairments and recovery following a rehabilitation treatment. Thirty-one chronic participants with ABI participated in a novel rehabilitation treatment composed of 22 sessions of a perturbation-based rehabilitation training. Dynamic balance was assessed using the Community Balance and Mobility scale (CB&M) and the 10-Meter Walking Test (10MWT). Brain function was estimated using resting-state fMRI imaging that was analysed using independent component analysis (ICA), and regions-of-interest analyses. Brain morphology was also assessed using structural MRI. ICA revealed a reduction in component-related activation within the sensorimotor and cerebellar networks post-intervention. Improvement in CB&M scale was associated with a reduction in FC within the cerebellar network and with baseline FC within the cerebellar-putamen and cerebellar-thalamic networks. Improvement in 10MWT was associated with baseline FC within the cerebellar-putamen and cerebellar-cortical networks. Brain volume analysis did not reveal structural correlates of dynamic balance, but dynamic balance was correlated with time since injury. Our results show that dynamic balance recovery is associated with FC reduction within and between the cerebellar and sensorimotor networks. The lack of global structural correlates of dynamic balance may point to the involvement of specific networks in balance control.

## Introduction

Acquired Brain Injury (ABI) is defined as damage to the brain that occurs after birth. Two common ABI conditions are Traumatic Brain Injury (TBI) and stroke^1^. Both etiologies are characterized by neuronal loss, and local and remote neurophysiological changes in structural and functional networks that are associated with the sensorimotor impairments and with the recovery process^2,3^. Furthermore, both etiologies trigger brain atrophy and neurodegenerative conditions^4,5^ that have been shown to be associated with motor, cognitive, and psychiatric decline^4,6^. Longitudinal studies indicate an association between brain atrophy post-stroke and gait-control decline^7,8^, as well as increased risk of motor-related neurodegenerative disorders (such as parkinson’s disease post-TBI)^9^.

Subjects post-ABI at the chronic phase frequently suffer from dynamic balance control impairments that restrict their mobility and limit their community integration and locomotor abilities^10–12^. Dynamic balance control is a complex function that is achieved by both feedback and feedforward control mechanisms and mediated by both central pattern generators at the spinal level and subcortical and cortical brain areas^13–15^. Imaging studies indicate the involvement of the cerebellum, basal ganglia and the motor cortex in gait and postural control in healthy subjects^16–19^. Lesions in the previously mentioned areas and in their inter-hemispheric connections are associated with gait and balance impairments^20–23^ and are being targeted for rehabilitation interventions in stroke and parkinson’s^24–26^.

Dynamic balance and mobility at the chronic phase of ABI have been demonstrated to respond to rehabilitation treatments such as virtual reality training, multi-disciplinary rehabilitation, and conventional balance training^27–30^. Furthermore, facing unpredictable perturbations, generated by mechatronic shoes, has been recently shown to be associated with improvement in dynamic balance^30^. Imaging studies from recent years indicate that cognitive and motor training effects can be predicted by functional brain connectivity reorganization and are associated with changes in functional connectivity^31–33^.

In this study, we investigate the neural correlates of dynamic balance impairments and recovery by longitudinally examining resting-state functional Magnetic Resonance Imaging connectivity (Rs-fMRI) in a group of ABI participants whose dynamic balance improved following a rehabilitation intervention^30^. Further, to map the neural correlates of dynamic balance impairments in ABI, we examine the correlation between dynamic balance impairments and the volume of pre-defined regions of interest in the cortex and sub-cortical regions^17–19,34^.

Specifically, we hypothesize that: (1) dynamic balance impairment will be associated with functional connectivity within and between neural networks that play a role in balance control, including the sensorimotor cortical network, the cerebellar network, and the basal ganglia network; (2) improvement in dynamic balance following training will be associated with reduction in connectivity between and within neural networks that play a role in balance control^24,26^; and (3) ABI-induced structural atrophy in networks that are involved in dynamic balance control will be associated with dynamic balance impairment and predictive of dynamic balance recovery.

## Methods

This study was approved by the Research Ethics Committee of the Reuth Rehabilitation Medical Center, Tel-Aviv, Israel and by the Research Ethics Committee of Soroka University Medical Center, Beer Sheva, Israel. All methods in this study were performed in accordance with the relevant guidelines and accordance with the declaration of Helsinki. All participants signed an informed consent prior to undergoing assessments. The ClinicalTrials.gov identifier number is NCT02215590, 13/08/2014.

### Subjects

#### Participants post-ABI

A total of 36 participants individuals (7 females, 29 males; mean age 60.44 ± 12.07 years) enrolled in the study. Participants were approached using a database of hospitalized patients at Reuth Rehabilitation Medical Center, Tel-Aviv, Israel and using an add that was published in a local newspaper. After signing an informed consent, participants went through a screening process that included a detailed neurological assessment of muscle strength and tone, sensation, reflexes, balance, and coordination assessments, in addition to a cognitive assessment and medical history review by a neurologist. Inclusion criteria were that participants: be within an age range of 18–80 years; have residual dynamic balance impairment due to ABI; be at least a year post-ABI (TBI or ischemic stroke) before recruitment; be able to walk at least 10 m with or without an assistive device; have no change in drug therapy for one month prior to trial and during the entire trial period; and lastly, score above 19 points on the Montreal Cognitive Assessment test (MoCA)^35^. Exclusion criteria were: presence of an acute progressive neurological, systemic, or musculoskeletal disorder affecting gait and balance; severe visual or hearing impairment; pulmonary or cardiac condition impairing exercise endurance; psychiatric disorders; and alcoholism or drug use.

### Experimental procedure

The study included behavioral and MRI assessments. Each assessment was conducted twice, pre- and post-intervention. Between the assessments, participants underwent a rehabilitation program using the Re-Step™ technology (mechatronic shoes)^29,30^.

Community balance and mobility scale (CB&M) was used to assess dynamic balance. This scale assesses difficulties in ambulation and balance skills needed for community integration, in individuals with stroke and adults with TBI^36,37^. The scale includes 13 tasks requiring multitasking and complex motor tasks (e.g., unilateral stance, forward-to-backward walking, descending stairs, and crouch and walk). Higher scores indicate better balance and mobility skills (maximum possible score = 96). Furthermore, we used the 10 Meter Walking Test (10MWT) for post-stroke participants in order to assess self-paced gait velocity^38^. The 10MWT was added to the behavioral assessments after the experiment had been started and was therefore performed only on the stroke participants.

### Outcome measures

Resting-state functional MRI connectivity, which shows sensitivity to changes in brain networks following brain injury^39^, and brain volume measure for detecting structural brain changes following brain injury^40^.

### Interventional procedure

22 sessions, held twice a week. Each session began with several warm-up exercises like mobilization and strengthening for 10 min, followed by training using mechatronic shoes that were developed for gait-rehabilitation of individuals with brain damage^29^. Each shoe has four pistons positioned at its sole that allow perturbation of gait by introducing sole-inclination changes during the swing phase.

Each training session with the shoes lasted 40 min and was followed by 10 min of cool-down exercises of stretching and relaxation (see,^30^ for details about the task and the perturbation protocol).

### MRI acquisition

Participants underwent two identical MRI sessions, pre- and post-intervention, each session including a 3D anatomical scan, Rs-fMRI scan, fMRI-Localizer scan, and DTI scan (not reported here).

High-resolution T1-weighted anatomical data were acquired with fast-spoiled gradient-echo (FSPGR) sequence, with a voxel size of 1 × 1 × 1 mm, (Repetition Time (TR) = 8165 ms, Echo Time (TE) = 3.74 ms, 256 × 256 acquisition matrix). The field of view (FOV = 192 mm) covered the entire cerebrum and the cerebellum. The duration of the scan was four minutes and 50 s. The fMRI data was acquired using a gradient echo EPI with voxel size of 3 × 3 × 3 mm (mm), TR = 2000 ms (ms), TE = 35 ms, flip angle = 77°, 35 slices, with a 0.6 mm gap, and lasted nine minutes and 50 s. The fMRI-Localizer scan was a block-design experiment containing six conditions (five movement-and one resting-condition); (1 + 2) left-and right-limb movements of dorsi/palmar flexion off palms, (3 + 4) left and right dorsi/plantar flexion for the ankles, and (5) bipedal ankle movements. The movement frequency of the limbs was equal to 1 Hz (Hz) and was demonstrated by the experimenter before the scan. Movement blocks of 12 s were separated by resting periods of 10 s, which were cued by a fixation cross [“+”] that was presented on a black screen. Visual cues for instructing hand and ankle movements were displayed on a screen during the experiment. Participants trained on the task before the scan using a dedicated apparatus located outside the scanner. The order of the blocks was random. In total, each localizer session included 25 movement blocks (five repetitions of each of the five movement conditions). This scan was conducted to define Regions of Interest (ROIs) based on functional activations.

The resting-state data acquisition parameters were similar to those in the fMRI-Localizer scan. During the resting-state session, a cross [“+”] was displayed in the middle of the screen, and participants were instructed to fixate on it during the scan. This scan lasted seven minutes and 26 s.

The magnetic resonance imaging and data acquisition were performed at the Imaging Center of Soroka Medical Center using a 3-Tesla Philips Ingenia whole-body MRI scanner (Philips Ingenia, Amsterdam, Holland).

### Imaging analysis

Functional, resting-state, and structural data were analysed by Brain Voyager 20.6 (Brain Innovation, Maastricht, The Netherlands). Brain segmentation was performed using FreeSurfer V 5.0 (developed by the Laboratory for Computational Neuroimaging at the Athinoula A. Martinos Center for Biomedical Imaging at Massachusetts General Hospital in Charlestown in Boston, MA).

### Pre-processing of the localizer scan data

Pre-processing included removal of the first two functional images of each run series to allow stabilization of the BOLD signal; correction of the slice scan time acquisition (ascending-interleaved, using a cubic-spline interpolation algorithm); and head-motion correction (using a trilinear/sinc interpolation) and a temporal high-pass filtering using a cut-off frequency of 2 sine/cosine cycles. Functional images were aligned to the T1-weighted structural image and incorporated into the 3D datasets through trilinear interpolation. Data was not spatially smoothed to maintain maximal sensitivity of the selected voxels to their selection criteria. Head motions during the scan was inspected during the analysis to verify lack of excessive and abrupt head motions (> 1 mm).

### Pre-processing of the resting-state scan data

The pre-processing included removal of the first two functional images of each run series to allow stabilization of the BOLD signal; correction for slice scan time acquisition (ascending-interleaved, using a cubic-spline interpolation algorithm); a trilinear interpolation approach in order to remove head motions; a high-pass (GLM-Fourier) frequency filter with a cut-off value of 2 sine/cosine cycles and a low-pass Gaussian-Full Width at Half Maximum (FWHM) of 1.9 data points^41^.

Further, in the ROI analysis we further used an 8th-order Butterworth filter with cut-off frequencies of 0.009 < *f* > 0.08 Hz^41^ before projecting out averaged signals of the white matter, the cerebro-spinal fluid, and head motion parameters (containing 6 regressors: translations and rotations in the x, y, and z dimensions). This step was conducted by running a GLM regression analysis. The residuals of this analysis were free from the unwanted components and were used as inputs in the resting-state functional connectivity (FC) ROI analysis. Functional connectivity was computed using a correlation analysis (Pearson correlation coefficient) between the time courses of pre-defined ROIs (M1, cerebellum, thalamus, putamen, superior frontal, and superior parietal).

Both the fMRI-task and Rs-fMRI data sets of each participant were spatially aligned onto the corresponding anatomical scan (T1 weighted structural scan) using an automatic alignment procedure (implemented in Brain Voyager 20.6). The results of the automatic alignment were inspected during processing and manually adjusted when necessary. Subsequently, the co-aligned images were transformed into Talairach space^42^.

### Definition of regions of interest

12 ROIs were examined: leg areas in M1 and cerebellum, thalamus, putamen, superior frontal, and superior parietal bilateral. The M1 and the cerebellar ROIs were identified using the localizer scan (contralateral ankle movement vs baseline, p < 0.05, cluster size > 1000). The coordinates of each ROI were selected based on activation peaks of the above contrasts and were obtained from the localizer scan. The thalamus, putamen, superior frontal, and superior parietal were anatomically defined for each participant using FreeSurfer V 5.0. The size of each ROI was defined as the number of functional voxels (3 mm isovoxel). [Detailed characteristics (mean, SEM) of each ROI are presented in Table 1].

**Table 1:** ROI Characteristics Presented as Means and SEM of Number of functional Voxels (3 mm isovoxel).

ROI	Mean	SEM
Right M1	1228.9	17.6
Right cerebellar	1158.4	19.1
Left M1	1189.8	21.6
Left cerebellar	1176	23.8
Left thalamus	6023.1	208.2
Left putamen	4325.3	191.9
Right thalamus	5353.03	194.3
Right putamen	4429.6	209.1
Left frontal superior	14,275.8	333.09
Left parietal superior	4805.6	150.4
Right frontal superior	13,587.5	342.06
Right parietal superior	4002.5	117.2

*ROI* region of interest, *M1* motor cortex, *SEM* standard error of the mean.

### Independent component analysis (ICA)

Resting-state data was analysed using ICA in Brain Voyager 20.6. This method allows the detection of a set of statistically independent spatial maps (networks) on a subject-by-subject basis during the resting-state scans and subsequently measures changes in the strength of these spatial maps after the intervention^43^. The analysis was composed of two stages. In the first, 30 ICA components were detected automatically in each scan based on the individual resting-state scans. In the second, consistent components within and across participants were automatically selected using the “self-organizing groups” feature in Brain Voyager that forms clusters (brain networks) across the brain according to similarity of their spatiotemporal structures^44^. The analysis was restricted to the sensorimotor network and the cerebellar networks that were shown to be sensitive to balance training^31^.

### Processing of the anatomical data for the volume analysis

3D anatomical scans were analysed using Free-Surfer software 5.0^45^.

### Statistical analysis

for statistical calculations, we used SPSS statistics (SPSS for Windows, Version 16.0; SPSS Inc., Chicago). The significance level was set to *p* < 0.05 for all statistical tests. Normality assumption was tested by using the Kolmogorov-Smirnova test (p > 0.05). Paired *t*-test was used for within-subject analyses of the behavioral data (pre-intervention vs. post-intervention). The ES (Cohen’s *d*) for the within-subject design was calculated by dividing the mean difference between pre- and post-intervention by the pooled SD.

For the resting state functional connectivity analysis, we used z-Fisher transformation in order to normalize the distribution of the correlation coefficients. The associations between functional connectivity measures, brain volume parameters and functional behavioral measures were assessed using multivariate linear regression models. To be specific, we ran regression models between the behavioral parameters: baseline CB&M, ΔCB&M, baseline 10MWT or Δ10MWT, and baseline FC or ΔFC which were computed between the ROIs. Post-hoc analysis was conducted on the significant regression models. The post-hoc analyses were not corrected for multiple comparisons. Whole-brain ICA was corrected for multiple comparison using a cluster-size correction for family-wise error rate at p < 0.05. The association between brain volume and dynamic balance was assessed using Pearson’s correlations.

## Results

Data from five participants from the ABI-group were excluded from the analysis for the following reasons: 1) One participant refused to participate in the MRI scan. 2) Four participants refused to continue with the intervention study due to loss of interest and transportation difficulties. The total number of participants included in the imaging analysis was 31 (11 = TBI and 20 = stroke). Additionally, one participant out of the 31 did not undergo the behavioural assessment post-intervention due to loss of interest.

The average age of the participants who participated in the study was 60.16 ± 12.85 (SD) years, with an average time since injury of 92.74 ± 144.43 (SD) months. The time interval between pre-and post-assessments was 117 ± 14.14 (SD) days (see Table 2 for additional information).

**Table 2:** Baseline Characteristics of each participant.

Participants	Age (year)	Gender	weight (kg)	height (cm)	Stroke/TBI	Time since injury, months (days)	Damaged hemisphere	MOCA score	Assistive walking device (no/yes)
S1	68	M	78	182	Stroke	99 (7)	R	24	No
S2	61	M	72	175	Stroke	31 (2)	L	26	No
S3	39	M	62	180	TBI	236 (2)	R	23	No
S4	63	F	57	155	Stroke	52 (6)	L	29	No
S5	69	M	89	182	Stroke	21 (28)	R	22	No
S6	69	M	73	172	Stroke	90 (29)	R	26	Yes
S7	61	M	69	186	Stroke	19 (2)	L	29	No
S8	68	M	62	161	Stroke	80 (4)	R	23	Yes
S9	61	F	53	163	TBI	435 (27)	L	25	Yes
S10	36	F	56	158	Stroke	20 (15)	L	28	No
S11	61	M	68	165	TBI	39 (11)	L	26	No
S12	69	M	81	169	TBI	563 (28)	R	25	No
S13	43	F	60	159	TBI	468 (5)	Bilateral	24	No
S14	69	M	88	175	Stroke	11 (2)	R	28	No
S15	72	M	90	170	Stroke	13 (2)	R	24	Yes
S16	47	M	100	172	Stroke	10 (21)	L	21	No
S17	53	M	81	175	TBI	24 (24)	Bilateral	23	No
S18	68	F	84	164	Stroke	14 (1)	L	30	No
S19	58	M	64	166	Stroke	25 (2)	L	25	Yes
S20	57	M	68	163	Stroke	14 (15)	L	21	No
S21	66	M	70	160	Stroke	14 (15)	R	27	Yes
S22	60	M	99	180	Stroke	14 (28)	R	22	No
S23	67	M	99	182	Stroke	22 (2)	R	26	No
S24	66	M	69	170	Stroke	25 (15)	R	24	No
S25	80	M	87	169	Stroke	23 (24)	R	23	No
S26	69	M	75	185	Stroke	226 (24)	R	21	Yes
S27	63	M	90	180	Stroke	24 (6)	R	28	No
S28	78	M	77	172	Stroke	12 (2)	R	23	Yes
S29	36	F	53	160	TBI	132 (5)	R	29	Yes
S30	63	F	68	157	TBI	43 (6)	R	25	No
S31	30	M	77	173	TBI	76 (14)	Bilateral	26	No

*F* female, *M* male, *TBI* traumatic brain injury, *R* right, *L* left.

### Recovery of dynamic balance and gait velocity following intervention

All 31 participants who were included in the imaging analysis completed the 22 sessions of training using the mechatronic shoes^29^. Dynamic balance (measures by CB&M scale) in ABI subjects (N = 30) was affected by the intervention. CB&M score changed from 37.73 ± 16.5 pre-intervention (T1) to 43.33 ± 17.01 post-intervention (T2) (higher scores indicating improvement) (*p* < 0.001) with a large effect size (ES = 0.83).

The 10MWT score for stroke participants (N = 21) changed from 0.88 m/s ± 0.34 pre-intervention (T1) to 0.95 m/s ± 0.32 post-intervention (T2) (*p* = 0.02) with a medium ES = 0.6^30^.

### Recovery was associated with a signal reduction in sensorimotor and the cerebellar networks

**Figure 1:**
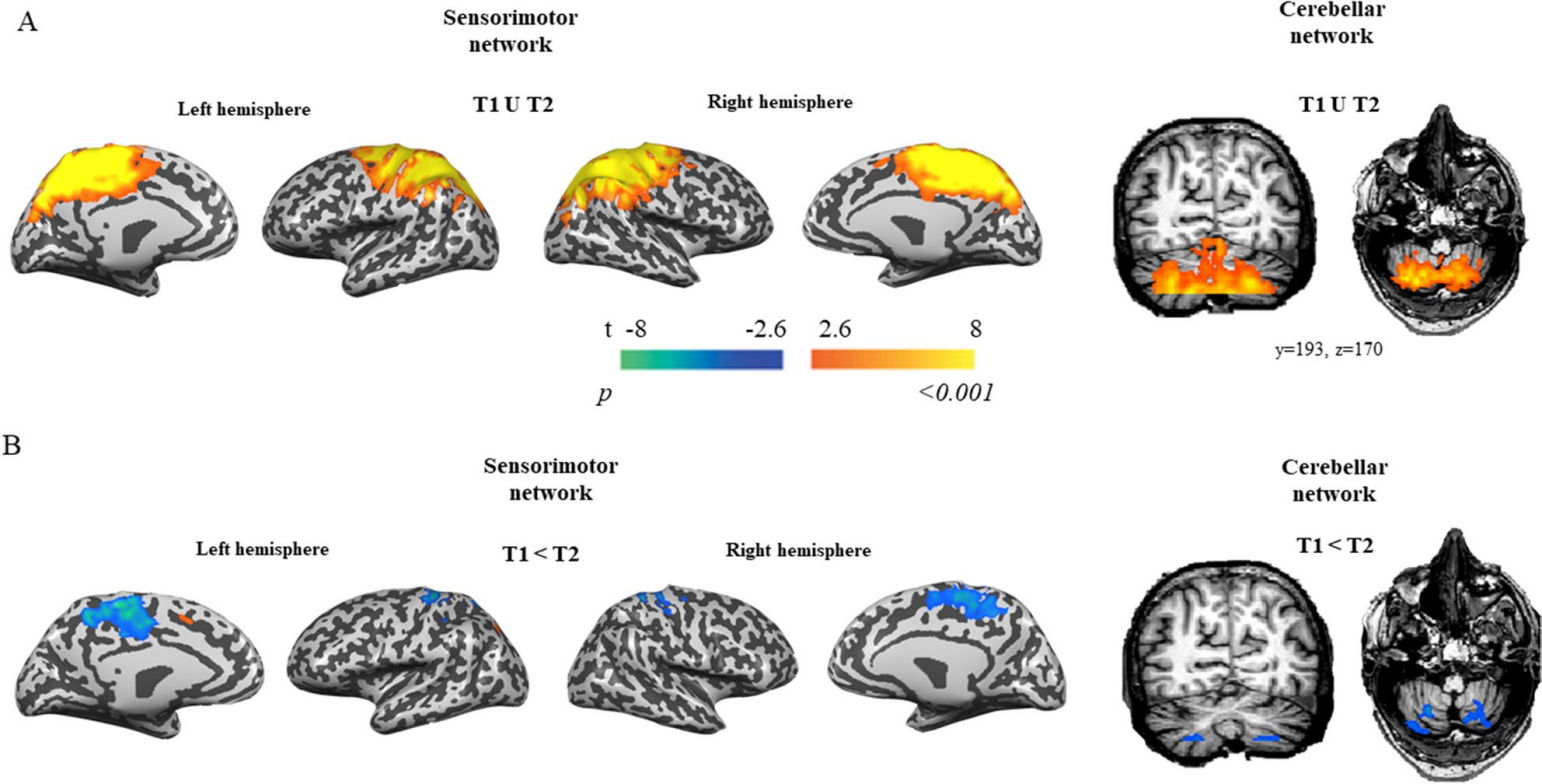
Two resting-state networks of interest identified by ICA: Sensorimotor (left) and Cerebellar (right). (**A**) Left, ICA component corresponding to the sensorimotor network. right, ICA component corresponding to the cerebellar network (transverse brain section-right figure), (coronal brain section-left figure). (**B**) Contrasts of both networks between pre (T1) and post-intervention (T2). Left, Sensorimotor network. Right, Cerebellar network (axial section (left) and coronal section (right). Color maps represent voxels’ significance. N = 31.

We first examined global changes in connectivity using an ICA approach. We focused our analysis on two resting-state spatial maps of interest that were shown to be involved in gait control^31^: the sensorimotor and the cerebellar spatial maps (Fig. 1A). For each network, we examined the strength of these networks at the voxel’s level using contrasts. This analysis revealed a reduced component-related activation in both networks (*p* < 0.05, cluster-size correction) (Fig. 1B). Increases in component-related activation following training were not found in both networks (*p* > 0.05).

### Recovery of dynamic balance was associated with reduced FC within the cerebellar network

Motivated by the ICA analyses, we aimed to better localize the networks that are associated with dynamic balance impairments and recovery. The first analysis focused on inter-hemispheric functional connectivity (IHFC) in the cerebellar and sensorimotor networks. Functional connectivity within these connections were not significantly modulated by the intervention. When searching for associations between connectivity and impairment and recovery, we found a significant association between ΔFC in the cerebellum and ΔCB&M [F (4, 25) = 3.41; β = − 0.57, *p* = 0.02 (uncorrected), R^2^ = 0.35] (Table 3).

**Table 3:** Multi-variate regression model of the association between ΔIHFC and recovery of dynamic balance (ΔCB&M).

Predictor	B	Standard error	Standardized β coefficients	t	*p*-value
(Constant)	6.45	1.15		5.58	0.001
ΔFC within the frontal superior lobes	3.18	3.15	0.17	1.01	0.32
ΔFC within the parietal superior lobes	1.38	2.49	0.1	0.55	0.58
ΔFC within the M1s	− 1.69	3.05	− 0.09	− 0.55	0.58
ΔFC within the cerebellum	− 11.20	3.46	− 0.57	− 3.23	0.003

*ΔFC* delta functional connectivity, *M1* motor cortex.

### Baseline resting-state Intra-hemispheric FC (IntraHFC) at the cerebellar-cortical and cerebellar-subcortical networks predicts dynamic balance recovery

Next, we examined the association between network connectivity within each hemisphere (IntraHFC) and dynamic balance and recovery. Here again, connectivity measures were not affected by the intervention. Regression models revealed the following:

(1) Baseline IntraHFC in the cerebellar-cortical and cerebellar-subcortical networks was associated with ΔCB&M [F (10, 19) = 2.27; *p* = 0.02, R^2^ = 0.58 (Table 4)]. Covariates contributing to the significant model were the left cerebellar-right thalamic network (β = − 0.51, *p* = 0.02, uncorrected) and left cerebellar-right putamen network (β = 0.62, *p* = 0.002, uncorrected).

**Table 4:** Multi-variate regression model of baseline IntraHFC at the cerebellar-cortical and cerebellar-subcortical networks to predict recovery of dynamic balance (ΔCB&M).

Predictor	B	Standard Error	Standardized β coefficients	t	*p*-value
(Constant)	3.96	1.43		2.75	0.01
FC between right cerebellum & left thalamus_T1	10.57	5.71	0.45	1.85	0.08
FC between right cerebellum & left putamen_T1	1.08	6.8	0.03	0.16	0.87
FC between left cerebellum & right thalamus_T1	− 12.03	4.97	− 0.51	− 2.41	0.02
FC between left cerebellum & right putamen_T1	13.64	3.69	0.62	3.68	0.002
FC between right cerebellum & left frontal superior_T1	− 7.34	5.11	− 0.26	− 1.43	0.16
FC between right cerebellum & left parietal superior _T1	3.01	6.85	0.09	0.43	0.66
FC between left cerebellum & right frontal superior_T1	− 4.61	3.03	− 0.23	− 1.52	0.14
FC between left cerebellum & right parietal superior _T1	− 1.58	1.21	− 0.20	− 1.29	0.2
FC between left cerebellum & right M1 _T1	6.97	3.46	0.38	2.01	0.05
FC between right cerebellum & left M1 _T1	7.52	6.07	0.2	1.23	0.23

*FC* functional connectivity, *T1* pre-intervention, *M1* motor cortex.

(2) Baseline IntraHFC in the cerebellar-cortical and cerebellar-subcortical networks was associated with Δ10MWT [F (10, 10) = 7.55; *p* = 0.002, R^2^ = 0.88 (Table 5)].

**Table 5:** Multi-variate regression model of baseline IntraHFC at the cerebellar-cortical and cerebellar-subcortical networks to predict recovery of self-paced velocity (Δ10MWT).

Predictor	B	Standard Error	Standardized β coefficients	t	*p*-value
(Constant)	0.06	0.01		3.74	0.004
FC between right cerebellum & left thalamus_T1	− 0.09	0.07	− 0.25	− 1.34	0.2
FC between right cerebellum & left putamen_T1	− 0.07	0.10	− 0.10	− 0.69	0.5
FC between left cerebellum & right thalamus_T1	− 0.03	0.06	− 0.09	− 0.57	0.5
FC between left cerebellum & right putamen_T1	0.15	0.05	0.44	3.06	0.01
FC between right cerebellum & left frontal superior_T1	0.2	0.07	0.47	2.85	0.01
FC between right cerebellum & left parietal superior _T1	0.3	0.08	0.55	3.75	0.004
FC between left cerebellum & right frontal superior_T1	− 0.05	0.03	− 0.18	− 1.57	0.14
FC between left cerebellum & right parietal superior_T1	0.08	0.01	0.69	5.59	0.0001
FC between left cerebellum & right M1 _T1	− 0.02	0.05	− 0.05	− 0.39	0.69
FC between right cerebellum & left M1 _T1	− 0.25	0.07	− 0.41	− 3.52	0.005

*FC* functional connectivity, *T1* pre-intervention, *M1* motor cortex.

Covariates contributing to the significant model were:

left cerebellar-right putamen network (β = 0.44, *p *= 0.01, uncorrected),

right cerebellar-left frontal network (β = 0.47, *p* = 0.01, uncorrected),

right cerebellar-left superior parietal network (β = 0.55, *p* = 0.004, uncorrected),

left cerebellar-right superior parietal network (β = 0.69, *p* < 0.001, uncorrected),

and left cerebellar-right M1 network (β = -0.41, *p* = 0.005, uncorrected).

### Brain volume analysis was not correlated with dynamic balance

Next, we searched for an association between structural parameters and dynamic balance measures using a multivariate linear regression model that included the following independent variables: (1) total gray matter volume, (2) total cortical WM volume and (3) left and right cerebellar WM volume; and baseline CB&M score as the dependent variable. The model revealed a non-significant association between brain volume and baseline CB&M score [F (4,26) = 0.6, *p* = 0.66]. To study the association between brain volume and potential recovery, we ran the same models but this time with **∆**CB&M as the dependent variable. This model was not significant as well [F (4,25) = 0.55, *p* = 0.69], suggesting that there is no linear dependency between the examined global structural brain variables and dynamic balance impairment and recovery.

**Figure 2:**
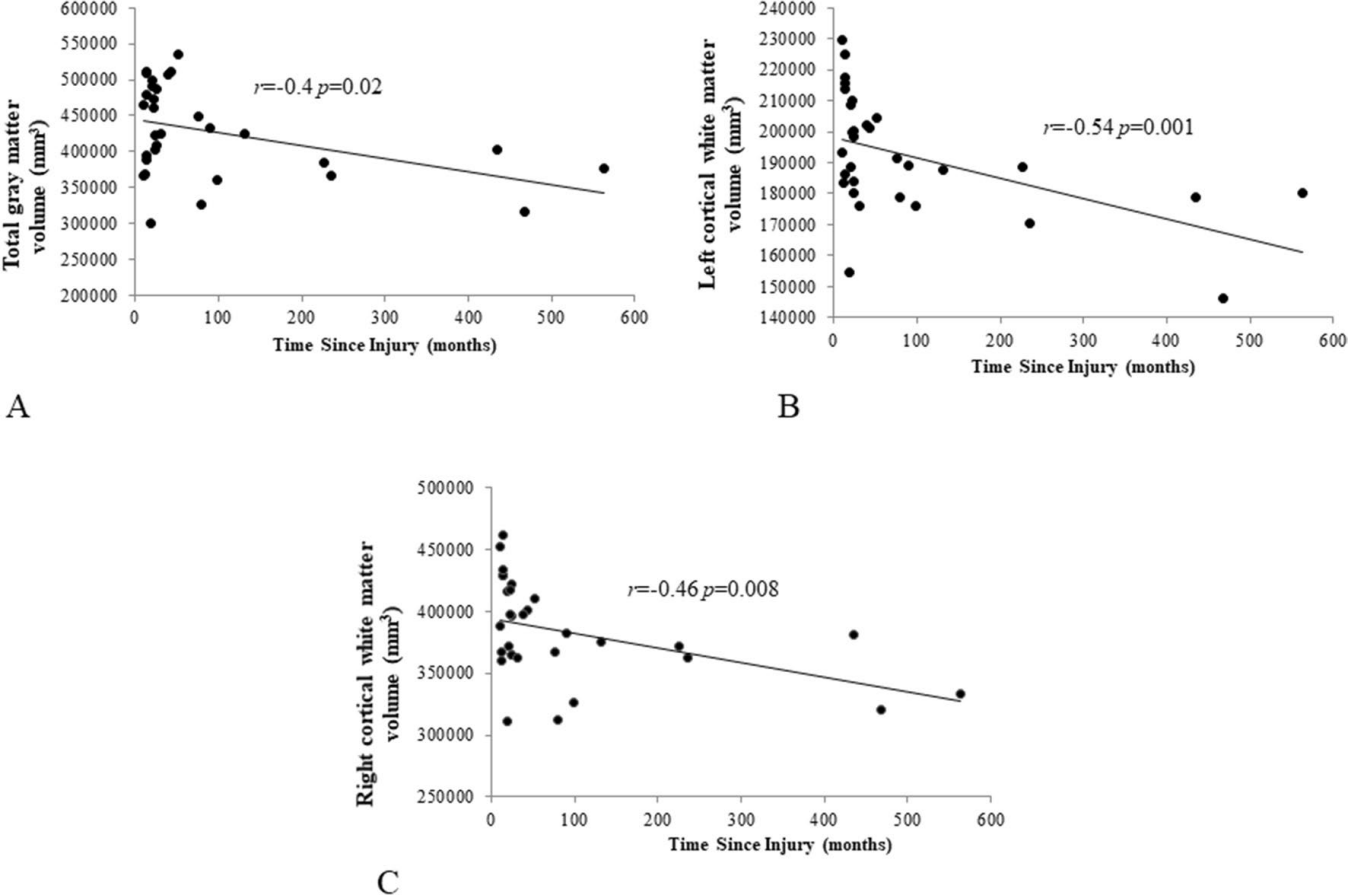
A scatter plot representing the dependence between brain volume and time since injury. (**A**) Total gray matter volume (mm^3^) and time since injury. (**B**) Left cortical white matter volume (mm^3^) and time since injury. (**C**) Right cortical white matter volume (mm^3^) and time since injury. Black line depicts the linear trend of the data. The range of time since injury range is between 10 and 563 months. *mm*^*3*^ = 3 cubic millimetres.

Lastly, in search of a possible degenerative mechanism for post-ABI dynamic balance impairment^2^, we examined the association between total gray matter (Fig. 2A) and cortical white matter (left and right hemispheres) (Fig. 2B,C) and time since the brain injury. The results revealed a significant association beween time since injury and these global ROIs (*r* = − 0.4 *p* = 0.02, r = − 0.54 *p* = 0.001, r = − 0.46 *p* = 0.008, respectively).

## Discussion

This study aimed to explore the neural substrates of dynamic balance by examining the association between dynamic balance impairments and recovery in chronic ABI participants and functional and structural brain measurements. The rationale for combining two subgroups of ABI (TBI and stroke) in the study was that both subgroups suffer from balance impairments due to a neuronal damage. Furthermore, both etiologies are characterized by initial neuronal loss, neurophysiological changes and disruptions of integration in structural and functional networks (diaschisis)^2^, resulting in sensorimotor impairments and loss of motor functions^2^. Lastly, in both groups, recovery is mediated by neural plasticity in intact cortical and sub-cortical regions^2^.

We report that dynamic balance recovery was associated with a reduction in connectivity in the sensorimotor and cerebellar networks. Furthermore, dynamic balance recovery was negatively associated with baseline connectivity within the cerebellar-thalamic network and with baseline connectivity in the cerebellar-M1 network. Dynamic balance recovery was also positively associated with baseline connectivity within the cerebellar-putamen network and both the cerebellar-frontal and cerebellar-parietal networks. We also found that while morphological features were not correlated with dynamic balance impairment and recovery, they were associated with the time that passed since injury.

### Network reduction as a biomarker of impairment post-ABI and of intervention-induced plasticity

Using the ICA approach, we found a reduction in component-related activation in the sensorimotor and cerebellar networks post-training. Furthermore, the results of the regression analysis showed that reduction in FC within the cerebellar network post-training and low baseline FC within the cerebellar-cortical and cerebellar-putamen were associated with better dynamic balance recovery. Therefore, we suggest that FC within the cerebellar-cortical and cerebellar-subcortical networks can provide insight into the neural substrates and mechanisms that support the recovery of dynamic balance. Here we propose that the reduced component-related activation and FC in sub-networks can be considered as a manifestation of increased network modularity (where the modules of the networks are the ROIs)^46^, which was described as the neural basis of complex behaviours in health^32,46^, and in disease^47,48^ and was shown to be associated with more flexible and adaptable behaviour, which is needed for benefit from training^32^. Future experiments should test this conjecture directly.

### The role of the cerebellar-cortical and cerebellar-subcortical in dynamic balance control

The resting state FC analysis revealed a correlation between dynamic balance recovery and the cerebellum, putamen, and thalamus. These inferred connections are consistent with the increasing evidence of the existence of subcortical loops that reciprocally connect the cerebellum with the putamen through the thalamus^49,50^, and functional interconnection between the cerebellum and the putamen^31^, the role of the putamen in gait and gait kinematics was demonstrated in studies in healthy^31,51^, and stroke participants^21,52^. Lastly, the resting-state FC analysis revealed a correlation between cerebro-cortical areas and dynamic balance recovery which is also consistent with previous studies highlighting the involvement of the cerebellum and the cerebral cortices in gait and balance control in healthy subjects^31,53^, and in those with post-brain injury^54,55^. Our results highlight the potential contribution of these cortical and subcortical brain areas to dynamic balance and gait-related recovery following brain injury.

### Chronicity of ABI and brain volume

Our findings exhibit a negative correlation between global brain volume reduction and time since injury, indicating a diffused atrophy that progresses with time. These findings are consistent with previous studies which showed that the spatial pattern of TBI-related atrophy affects multiple grey and white matter areas^35,56^ and also are consistent with a previous longitudinal study demonstrating that enhanced reductions in total brain volume with time reduced white matter integrity post-ABI^56^. Importantly, we did not find a direct association between brain atrophy and dynamic balance. The lack of association can be explained by the involvement of multiple brain areas in dynamic balance control and the variability among subjects in terms of damage and/or recovery mechanisms. Thus, there might be an indirect association between brain atrophy and dynamic balance which is manifested by the alteration in functional networks, as has been reported previously in cognitive impairments post-stroke^57^. Furthermore, since atrophy is a process associated with time, conducting longitudinal assessments over time will provide a better understanding of the relationships between atrophy and dynamic balance.

### The integration of structural and functional brain measures (the multimodal approach)

Brain functional connectivity and structural morphology are clearly not independent. Two emerging perspectives in the neuroimaging literature attempt to explain the association between structural damage and network function. Firstly, studies indicate a negative association between the extent of the brain damage (post-stroke/TBI/Tumours) and FC depicting a depression of neural activity in brain regions remote from the initial site of brain damage due to reduced FC (diaschisis)^3,58^. Secondly, studies report an increase in FC within brain regions that had reduced structural connectivity, which may represent the reorganization of the system following the brain damage^57,59^.

Future studies should adapt a multi-modal approach of brain network assessments post brain damage, as this approach can open new perspectives into the consequences of brain damage and its impact on impairment and recovery.

Several limitations of this study should be acknowledged:

(1) Resting-state data was collected only for post-ABI with no control reference. This limitation confines the discussion about abnormal patterns of connectivity but does not affect the interpretation of the longitudinal data. (2) The absence of follow-up assessments limits the estimation of the efficacy of the treatment and its long-term neural outcomes. (3) There is an increase in the risk of type-1 bias due to multiple comparisons, especially in the post-hoc analysis of the regression models that were not corrected for multiple comparisons. We suggest taking these results as preliminary exploratory evidence and call for their replication. (4) While all subjects suffered from dynamic balance impairment due to ABI, the different etiologies (TBI and stroke) increased the inter-subject variability with respect to the location of damage. (5) We could not reproduce the ICA results with the ROI analysis. This might be due to the different methodologies and the procedure that were adopted in order to define the ROIs.

In conclusion, functional and structural mapping of brain networks reveals widespread alterations of networks following brain injury and rehabilitation. We suggest that these alterations were more likely to have resulted from the training than from the chronic time post-injury, since spontaneous changes are less prominent at the chronic phase post brain injury. Our study demonstrates functional connectivity changes in the cerebellar and sensorimotor networks following a rehabilitation treatment for post-ABI participants and suggests neural markers for the treatment’s gains. The lack of inter-subject correlations with structural atrophy suggests that dynamic balance is an emergent feature of functional and composite networks. Our results contribute to the understanding of the neural correlates of dynamic balance and depict several markers of recovery that should be further investigated.

## Abbreviations

ABI: Acquired brain injury
CB&M: Community balance and mobility
FC: Functional connectivity
IHFC: Inter-hemispheric functional connectivity
IntraHFC: Intra-hemispheric functional connectivity
MoCA: Montreal cognitive assessment
ROI: Regions of interest
Rs-fMRI: Resting state functional magnetic resonance imaging
TBI: Traumatic brain injury
TE: Echo time
TR: Repetition time
10MWT: 10 meter walking test
